# Iranian traditional medicinal plants for management of chronic heart failure: A review

**DOI:** 10.1097/MD.0000000000033636

**Published:** 2023-05-12

**Authors:** Faeze Keihanian, Mohsen Moohebati, Amin Saeidinia, Seyed Ahmad Mohajeri

**Affiliations:** a Pharmaceutical Research Center, Pharmaceutical Technology Institute, Mashhad University of Medical Sciences, Mashhad, Iran; b Cardiovascular Department, Faculty of Medicine, Mashhad University of Medical Sciences, Mashhad, Iran; c Pediatric Department, Akbar Hospital, Faculty of Medicine, Mashhad University of Medical Sciences, Mashhad, Iran.

**Keywords:** chronic heart failure, heart failure comorbidity, medicinal plants, novel drug, traditional medicine

## Abstract

Chronic heart failure is a public health problem with a high prevalence worldwide and an important topic in clinical cardiology. Despite of advances in the drug treatment strategy for heart failure, the number of deaths from this condition continues to rise. It will be a renewed focus on preventing heart failure using proven and perhaps novel drugs. Management will also focus on comorbid conditions that may influence the progression of the disease. Traditional medicine has a potential to introduce different approaches for treatment of some disorders. We here reviewed top medicinal plants, according to traditional medicine to experimental studies, and their potency for the treatment of chronic heart failure based on the evidence of their functions.

## 1. Introduction

Prior to setting up current modern medicine, humans used different methods like herbal medicine. Medicinal plants are the origination of various commercial agents used today in clinical practice.^[1]^ Although there is general acceptance toward using medicinal plants in the majority of the world, there is a little evidence with well-designed studies. There are many promising candidate agents for some disorders that are failed to be cured by routine medicine. Because of the optimistic view of people toward herbal medicine, they use regardless of its probable adverse or beneficial effects.^[2–4]^ The World Health Organization has also suggested the evaluation of medicinal plants in different disorders for which there are few safe modern drugs.^[5,6]^ The interest of the medical healthcare system is to utilize natural products in disease management. Medicinal plants are the source of novel candidate therapeutic options in different disorders.^[7]^ There are some data related to ethnopharmacology that emphasize using medicinal plants in traditional medicine and can make ideas in novel drug development.^[8]^ Focus on the discovery of new drugs with the most minor complications and best compatibility is going on.^[9]^

The burden of chronic diseases, among which cardiovascular disease (CVD) is increasing rapidly worldwide.^[10–12]^ Cardiovascular disease is a compound for clusters of disorders afflicting the heart and blood vessels. They include hypertension, coronary heart failure, stroke, and heart failure (HF).^[10,13,14]^ CVD is assuming an increasing role as a significant cause of morbidity and mortality.^[15]^ About 17.3 million deaths were estimated because of CVD by World Health Organization in 2013,^[15]^ and it has forecast to increase to 23.3 million in 2030. Different habits and unhealthy practices have been termed predisposing factors to CVD; this includes high intake of saturated fat and cholesterol,^[16]^ stress, cigarette smoking, physical inactivity, and diabetes,^[13]^ atherosclerosis, and hypertension.^[17]^ The pathogenesis of CVD through these factors is by causing oxidative stress, which is characterized by an upsurge in reactive oxygen species beyond the threshold of the endogenous anti-oxidant system,^[18]^ endothelial dysfunction,^[19]^ or alteration in the vasculature/vascular injury resulting in the mobilization of inflammatory markers.^[20]^ Commonly used conventional drugs in the treatment of CVD are with side effects^[21]^ and are very expensive, hence the need for a safer, cheaper, and more potent alternative.^[15]^ Chronic heart failure (CHF) is a general health issue in the world, and a serious subject in clinical cardiology,^[22]^ and its prevalence is more than twenty-three million over the world.^[22]^ It was estimated that 0.3% of admission etiologies were related to HF in an observational study in Iran.^[23]^ The management of CHF is combined options including beta-blockers, digitalis, angiotensin-converting enzyme inhibitors or angiotensin receptor blockers, diuretics, aldosterone receptor antagonists, and vasodilators, based on international guidelines.^[24,25]^ Although there are many improvements in HF management, mortality-related cases are increasing.^[26]^ There is a need to evaluate the proven or probable new drugs for the management or prevention of HF. This new strategy is also better to affect comorbidities and decrease disease progression.^[27]^ Traditional medicine has a good potential source for introducing new drugs.^[28,29]^ This traditional medicine includes a group of experiences used in the diagnosis, prevention, and management of disorders.

In this review, we assessed different medicinal plants based on traditional medicine usage, experimental investigations, and their capacity to manage CHF. We explored various basic and clinical studies in EMBASE, Science Citation Index, TRIP, Database of Abstracts of Reviews of Effectiveness (DARE), MEDLINE, Cochrane Library, NHS, and Social Science Citation Index. We also assessed the literature on traditional medicine, and each plant’s characteristics were extracted and mentioned in this review according to components of CHF’s treatment.

## 2. Berberis vulgaris

It is a medicinal plant with ancient utilization history.^[30]^ The plant is a bush 1 to 3 meters in height, spiny, with yellow wood and long leaves.^[31,32]^ Different pharmacological investigations have expressed the cardiovascular effects of berberine and *Berberis vulgaris (B vulgaris*), including prohibiting ischemia-induced ventricular tachyarrhythmia, advancement of cardiac contractility, and lowering peripheral vascular resistance and blood pressure.^[33–35]^

### 2.1. Chemical constituents

*B vulgaris* has several active constituents; the most important of them are oxyacanthine, berberine, and other alkaloids like malic acid, columbamine, jatrorrhizine, berbamine, and berberubin.^[36]^

### 2.2. Inotropic effect

Parsaee et al^[36]^ showed a potent inotropic effect but not chronotropic for a hydro-alcoholic extract of Berberis vulgaris on isolated-rabbit-heart without tachycardia which was mainly related to calcium channel opening effect or intracellular cyclic adenosine monophosphate (cAMP). It can elevate the contractile answer by that enhancement of cardiac pumping performance. They showed that it did not affect on the β-adrenoreceptor. The impact of the hydroethanolic extract on heart rate was the same, but cardiac contractility was different from diltiazem. It had a positive effect on heart contractility in a concentration-dependent effect matter. It is demonstrated that high concentrations of berbamine can inhibit cardiac muscle contraction in isolated rabbit hearts.^[37]^ This effect probably contributed to the inhibition of calmodulin.^[38,39]^ In an investigation, a long-term hypotensive impact made by the intravenous injection of 10% infusion of the extract has led to a direct myotropic impact.^[40]^ In an in vitro study, the effects of berberine on the physiologic characteristics of myocardium in guinea pigs were evaluated. It was shown that 0.1-300 μmol/L berberine made a positive inotropic and negative chronotropic action in a dose-dependent pattern.^[41]^ Zhang et al demonstrated that the positive inotropic effect of berbamine can be related to increasing myofilament ionized calcium sensitivity, preventing the complications of different cardio-tonic drugs that enhance heart contractility by raising intra-cellular ionized calcium levels. This response seems to be due to increasing cytosolic protein kinase C. They also indicated that a higher level of berbamine (300 nano-molar) can be related to a negative inotropic impact by decreasing ionized calcium transients and cellular shortening, thus recommending a bi-phasic concentration-dependent adjustment of cardiac performance.^[42]^

### 2.3. Vasodilator effect

Fatehi-Hassanabad et al^[33]^ demonstrated that various concentrations of the aqueous extract of Berberis vulgaris extract (0.5–10 mg/kg) decreased the contractile effect of phenylephrine. Choiu et al^[43]^ revealed in their study that berberine made dose-dependent vasodilation, and the vaso-relaxant impact of berberine was weakened by a lack of endothelium. They showed that 2 known inhibitors of endothelium-derived relaxing factor, L-NG-nitro arginine (L-NOARG) (a specific inhibitor of nitric oxide formation from L-arginine) and methylene blue (an inhibitor of soluble guanylyl cyclase), markedly weakened the vaso-dilator response to berberine. Berberine also can significantly inhibit the phenylephrine-induced phasic contraction, against verapamil, without effect on the high potassium-induced contraction. Another finding of berberine was inhibiting of the caffeine-induced contraction in a Ca2+-free/EGTA medium. Parsaee et al^[36]^ demonstrated a vasodilatory effect of the hydroalcoholic extract of the herb. It is related to decreasing Ca2 + concentration. Ko et al^[44]^ indicated that berberine could affect both endothelium and the underlying vascular smooth muscle to make relaxation. Nitric oxide from endo-thelium might be firstly responsible for the berberine-induced endothelium-dependent relaxation. At the same time, activation of tetramethylammonium-, 4-aminopyridine- and Ba2+-sensitive K + channels, inhibition of intracellular ionized calcium released from caffeine-sensitive pools, or a direct relaxant effect, is likely account for the berberine-induced endothelium-independent relaxation. Xu et al^[45]^ mentioned in their study that berberine had blockade effects on both L-type and T-type calcium channels. Bova et al^[46]^ explained the vasorelaxant effect of Berberine in their research.

They showed that berberine discouraged the answer of the guinea pig aortic strips to nor-epinephrine and histamine but did not lower the high K^+^-evoked level. Berberine reduced both the rate and the relative contribution to the developed tension of the initial, rapid phase. Berberine prevented the answer of aortic strips incubated in Ca^2+^ to nor-epinephrine but did not decrease caffeine-induced-contraction and inhibited phospholipase-C-activated contractile response, which has been attributed to the output of inositol-phosphate-3 in smooth-muscle cells. They also showed in their in vitro study that Berberine could not affect the production of inositol-phosphates activated by Arg-8-vasopressin. In Wong’s investigation,^[47]^ the mechanism of the aortic relaxation-induced by a low level of berberine was evaluated in the isolated aorta of the rats. It was shown that the mechanism of the aortic relaxation induced by a low berberine concentration is dependent on the existence of endothelium. Marin Neto^[35]^ demonstrated that the low-dose-infusion made no considerable circulatory alterations, regardless of the reduction in heart rate. He showed decreases in systemic and pulmonary vascular resistance and in right atrial and left ventricular end-diastolic pressures; improvement in cardiac index, stroke index, and LV ejection-fraction measured by contrast angiography; increases in hemodynamic and echocardiographic indices of LV function.

### 2.4. Antihypertensive component

It is shown that aqueous extract of *B vulgaris* can lower the systemic arterial blood pressure, in rats.^[33]^ Aqueous and ethanolic extracts of *B vulgaris* can inhibit angiotensin-converting enzyme (ACE) with 65% and 23% inhibitory effect, respectively in vitro.^[48]^ In another study, it was shown that administration of the *B vulgaris* extract (0.5–10 mg/kg) markedly decreased the mean arterial blood pressure and heart rate in anesthetized normotensive and desoxycorticosterone acetate-induced hypertensive rats in a dose-dependent pattern.^[49]^ Berberine in *B vulgaris* can block alpha-1-adrenoreceptor in the aorta of rats.^[50]^ Chun et al showed that intravenous infusion of berberine decreased the blood pressure and the heart rate of rats, most likely by inhibiting true cholinesterase and potentiation of acetylcholine levels.^[34]^ Kang et al^[51]^ examined the relaxant and anti-constrictive effects of berberine in the isolated thoracic aorta in rats. They showed that intravenous injection of berberine decreased the mean arterial pressure of anesthetized rats in a dose-dependent pattern. The ACE activities were prevented markedly by adding berberine in a dose-dependent way of which the inhibitory concentration of 50% (IC50) value of berberine for ACE was 42 μg/mL. In the endothelium-intact rings, angiotensin I-induced contraction was markedly attenuated by prior exposure of aortic rings to berberine. They showed that it could increase the NOx and cGMP productions and affect the contraction process in the vascular tissues. Lee et al^[52]^ tested the mechanisms of berberine in inhibiting vascular smooth muscle cell proliferation as it did in endothelial cells or cancer cells. Their results indicated that berberine markedly prevents growth factors, mainly angiotensin II (AngII) and heparin-binding epidermal growth factor, induced vascular smooth muscle cell proliferation and migration in vitro, and this impact is obtained by delaying or partially suppressing activation of Akt pathway rather than ERK pathway. The summary of *B vulgaris* effects is listed in Table 1.

**Table 1:** Potential effects of *B vulgaris* in treatment of CHF.

Study	Therapeutic component	Dose/active constitute/extract	Type of study	Major findings/mechanism
Parsaee et al^[38]^	Inotropic	Hydro-ethanolic extract berbamine	In vitro isolated rabbit heart	Improving cardiac pumping function calcium channel opening effect or intracellular cAMP inhibition of calmodulin
Wang et al^[43]^	Inotropic	Berberine 0.1–300 μmol/L	In vitro study myocardium of guinea pig	Positive inotropic and a negative chronotropic
Zhang et al^[44]^	Inotropic	Berbamine	In vitro study	Positive inotropic effect increasing myofilament Ca^+2^ sensitivity, intracellular Ca^+2^ concentration, cytosolic protein kinase C
Fatehi-Hassanabad et al^[35]^	Vasodilator	Aqueous extract 0.05–1 mg/100 g body weight	In vivo study rats	Reduced the contractile effect of phenylephrine
Choiu et al^[45]^	Vasodilator	Berberine	In vitro study	Vasodilatory effect is mediated by endothelium-derived relaxing factor (EDRF), nitric oxide formation, soluble guanylyl cyclase Berberine significantly inhibit the phasic contraction
Ko et al^[46]^	Vasodilator	Berberine	In vitro study	Endothelium and vascular smooth muscle relaxation by Nitric oxide and intracellular Ca^2+^ release from caffeine-sensitive pools
Xu et al^[47]^	Vasodilator	Berberine	In vitro study guinea pig ventricular myocytes	Blocking effects on both L- and T-type calcium channels
Bova et al^[35]^	Vasodilator	Berberine	In vitro study guinea pig aortic strips	Inhibited the response of the guinea pig aortic strips by production of inositol phosphate-3 in smooth muscle cells
Wong^[48]^	Vasodilator	Berberine	In vitro study rat isolated aorta	Its effect is dependent on the presence of endothelium
Fatehi-Hassanabad et al^[35]^	Antihypertensive	Aqueous extract	In vivo study rats	Reduce the systemic arterial blood pressure
Ziai et al^[50]^	Antihypertensive	Aqueous and ethanolic extracts	In vitro study	Inhibit ACE enzyme
Fatehi et al^[34]^	Antihypertensive	Aqueous extract 0.05–1 mg/100 g body weight of rat	In vivo study rats	Reduce the mean arterial blood pressure and heart rate
Chun et al^[52]^	Antihypertensive	Intravenous infusion of berberine	In vivo study rats	Lowered the blood pressure and the heart rate by inhibition of true cholinesterase and potentiation of acetylcholine levels

ACE = angiotensin-converting enzyme, cAMP = cyclic adenosine monophosphate, CHF = chronic heart failure.

### 2.5. Other beneficial components

Ischemic pre-conditioning is a firm adjustable answer, which prevents the heart from subsequent ischemic injury,^[53]^ which is mediated by intracellular Ca^+2^ overload leading to contractile dysfunction.^[54]^ Berbamine in the berberis genus, can inhibit calpain by preserving Ca^+2^ hemostasis and activate phosphoinositide-3-kinase and protein-kinase-B enzymes that lead to inhibition of the glycogen-synthetase-kinase-3β and opening the mitochondrial ATP-sensitive K-channels.^[55]^ It is demonstrated that Berbamine can enhance post-ischemic cardiac performance in a concentration-dependent pattern.^[55]^ Berberine, another constituent of the berberis genus, can also inhibit cardiac hypertrophy in rats (received high doses of L-thyroxine or underwent surgical-binding of the aorta) by increasing cardiac NO level, Na^+^/K^+^ ATPase and Ca^+2^ ATPase activities, besides diminishing plasma levels of nor-epinephrine and controlling the sympathetic tone.^[56]^ Zeng et al^[57]^ assessed the efficacy and safety of berberine for chronic congestive heart failure (CHF). Their study was performed on 156 patients with CHF and more than ninety ventricular pre-mature-contraction and, or non-sustained ventricular-tachycardia on twenty-four-hour Holter monitoring that were randomly allocated into 2 groups (conventional therapy for CHF + berberine 1.2 to 2.0 g/daily (n = 79) versus conventional therapy + placebo (n = 77)). There was a marked increase in exercise capacity, left ventricular ejection fraction, dyspnea fatigue index and lowering times, and complexity of ventricular pre-mature-contraction in comparison with the control group after 8 weeks and average twenty-four months follow up. A considerable decrease in mortality were seen in the berberine-treated group within long-term follow up.

Meng et al^[58]^ tested the probable effects of berberine on 130 acute coronary syndrome patients following Percutaneous Coronary Intervention (61 patients treated with berberine (300 mg, 3 times a day, for thirty days) plus standard treatment versus patients with alone standard treatment. Their findings demonstrated that in the berberine group, matrix metalloproteinase (MMP)-9, ICAM-1, vascular cell adhesion molecule (VCAM)-1, C-reactive protein, interleukin-6 and monocyte chemoattractant protein-1 were markedly decreased rather than base indexes, and another group. VCAM-1 and inter-cellular adhesion molecule (ICAM-1) are 2 groups of the immune-globulin gene super-family that had significant and various roles in the adhesion of blood cells to the vascular-endothelium. The importance of these molecules in the process of athero-genesis is validated by their immune-histochemically approved higher expression of the athero-sclerotic plaques^[59,60]^ and by in vitro models revealing a delayed-lesion development in their lacking.^[61]^ VCAM-1 serum levels strongly estimate the higher risk of subsequent cardio-vascular complications and spread the prognostic data obtained from classic biochemical markers.^[62]^ Other beneficial effects were inducing a significant decrease in low-density-lipoprotein-cholesterol and triglycerides than standard treatment lonely, with no impact on high-density-lipoprotein-cholesterol and severe complications of berberine.

## 3. Berberis integerrima

*Berberis integerrima* Bunge belongs to Berberidaceae, famous as an antidiabetic plant in Persian folk medicine based on an ethnopharmacology study.^[63]^ In contrast to Berberis vulgaris broad spectrum of research and evidence, data on *B integgrima* is not very well described yet. In traditional medicine references, the potential diuretic effect and antihypertensive effect of this medicinal plant are reported.^[32,64]^

### 3.1. Chemical constituents

It is shown by chromatography that reticuline, isoboldine, isocorydine, glaucine, armepavine, oxyacanthine, heliamine, intebrinine (IV), and intebrimine are major compounds of different fractions of *B integgerima*.^[65]^ Another important compound in B. integerrima is anthocyanin. In a study, total anthocyanin content in *B integerrima* fruits was 863.26 ± 1.37 mg in 100 g ethanolic extract and 812.03 ± 23.16 mg in 100 g aqueous extract.^[66]^

### 3.2. Antihypertensive component

It is shown in different studies that the extract of *B integerrima* can inhibit the ACE enzyme. The percent of inhibition was reported 88.2 ± 1.7%,^[67]^ 81%^[48,68]^ and 80%,^[69]^ which were the high results for ACE inhibition. Mahdavi et al^[70]^ examined the effect of *B integerrima* fruit on monocrotaline-induced pulmonary hypertension in vivo. Their results showed that in comparison with the M group (monocrotaline), *B integerrima* (200 mg/kg) or sildenafil markedly decreased the right ventricular systolic pressure, right ventricular hypertrophy, and the medial wall thickness with no effect on the plasma level of endothelin-1, glutathione peroxidase, and the malondialdehide of the lung. They demonstrated that this effect was more potent than the sildenafil and might have been interfered with by mechanisms other than the regulation of the endothelin-1 or redox system. Some different spontaneously hypertensive rat models showed the effects of various types of anthocyanin in lowering systolic blood pressure.^[71–73]^ The high content of anthocyanin in *B integerrima* extract can be related to its potency in CHF management components. Anthocyanins are glycosylated polyhydroxy and polymethoxy derivatives of flavilium salts and are members of the flavonoid family, having a feature of C3–C6–C3 carbon structure.^[74]^ It is mentioned that anthocyanins can have positive effects on the cardiovascular system by nitric oxide metabolism in endothelium and heart tissue, Monocyte chemotactic protein 1 (MCP-1), TNFα, MMP-1, MMP-9, and IL-10 (chemotaxis), inhibition of NF-κB activation (signal transduction pathways), suppress the induced secretion of several adhesion molecules and decrease in serum CRP.^[75]^

### 3.3. Vasodilator component

It is shown that anthocyanin extracts had endothelium-dependent relaxation capacity in porcine coronary arteries.^[76]^ It also can increase cardiac glutathione concentrations in rats, leading cardioprotection.^[77]^ Pelargonidin, as an anthocyanin inhibits iNOS protein and mRNA expression as well as the NO production in a dose-dependent manner in macrophages exposed to lipopolysaccharide (LPS). Pelargonidin also can inhibit the activation of NF-κB, which is a significant transcription factor for iNOS.^[78]^ Other studies mentioned endothelium-dependent vasodilation of different anthocyanins.^[79,80]^ The summary of *B integerrima* effects is listed in Table 2.

**Table 2:** Potential effects of *B integerrima* in treatment of CHF.

Study	Therapeutic component	Dose/active constitute/extract	Type of study	Major findings/mechanism
Ziai et al^[50]^	Antihypertensive	Aqueous extract	In vitro study	Inhibit ACE enzyme
Mahdavi et al^[72]^	Antihypertensive	200 mg/kg	In vivo monocrotaline-induced pulmonary hypertension	Reduced the RVSP, RVH, and the medial wall thickness
Bell et al^[78]^	Vasodilator	anthocyanin	In vitro	Endothelium-dependent relaxation in porcine coronary artery
Hamalainen et al^[80]^	Vasodilator	Pelargonidin	In vitro	Inhibits iNOS protein and mRNA NO production, inhibit NF-κB activation

ACE = angiotensin-converting enzyme, CHF = chronic heart failure, iNOS = inducible nitric oxide synthase, NF-κB = nuclear factor kappa-light-chain-enhancer of activated B cells, RVH = right ventricular hypertrophy, RVSP = right ventricular systolic pressure.

### 3.4. Other beneficial components

In another investigation on isoproterenol-induced postinfarction remodeling in rats, an alcohol-free red wine containing anthocyanin showed a protective effect on hearts by repressing hypertrophy-associated increased phosphorylation of protein kinase C α/β II and by activating Akt/protein kinase B (Akt).^[81]^ Anthocyanins affect on cholesterol distribution, protecting endothelial cells from CD40-induced proinflammatory signaling.^[82]^ Some anthocyanins showed an inhibitory effect on LPS-induced nitric oxide biosynthesis in vitro.^[83]^ It has been shown that anthocyanin delphinidin decreases the extent of both necrotic and apoptotic cell death in cultured cardiomyocytes and reduces infarct size after ischemia in rats. Both effects were through inhibition of STAT1 activation.^[84]^ Joukar et al^[85]^ assessed the influence of *B integerrima* aqueous extract on the hemodynamic and the electrocardiogram indices of rats for 15 days. They had showed that different doses of barberry fruit extract had no significant effect on blood pressure, heart rate, RR interval, P duration, and Q wave amplitude of electrocardiogram. Their results suggested that high doses of barberry extract definitely prolong the depolarization phase and shorten the repolarization phase of ventricular muscle and hence induce alteration in heart electrical conductivity.

## 4. Apium graveolens

*Apium graveolens* is a medicinal plant in traditional medicine with numerous health benefits. In recent pharmacological studies, celery has demonstrated antioxidant, hypolipidemic, and anti-inflammatory activities.^[86,87]^ Its seed was also administered as an antihypertensive agent,^[88,89]^ diuretic agent,^[31,32,64]^ and cardiotonic agent^[64]^ in folk medicine. These positive components predispose this plant for introduction as a therapeutic agent for CHF. Pharmacological evidence will be discussed in continue.

### 4.1. Chemical constituents

The seed of *A graveolens* has limonene as a major constituent. It also contains a-p-dimethyl styrere, N-pertyl benzene, caryophyllene, a-selinene, N-butyl phthalide, sedanenolide along with sablnene, b-elemne, trans-1,2-epoxy limonene, linalool, isovalaric acid, cis-dihydrocarvone, trans-dihydrocarvone, trepinene-4-ol, 1-cis–p-menth-2,8-diene-1-ol, trans-p-menth-2,8-diene-1-ol, alpha-terpineol, carvone, trans-8-diene 1-ol, perialdehyde, and thymol.^[90,91]^

### 4.2. Vasodilator component

Zhang et al^[92]^ determined the relaxant effect of apigenin, a compound of *A graveolens*. They showed that apigenin relaxed the phenylephrine-precontracted endothelium-intact aortic rings with an IC50 value of 3.7 ± 0.5 μM, and removal of a functional endothelium significantly attenuated this relaxation. They suggested that apigenin did not influence protein kinase C-mediated contractile mechanisms in rat aorta but did on acetylcholine on phenylephrine-induced contraction, which was due to an increase in the guanosine 3′,5′-cyclic monophosphate (cGMP) content of endothelium-intact tissues. They revealed that besides the influx and release of Ca^+2^ nitric oxide (NO), and cGMP might account for the apigenin-induced endothelium-dependent relaxation and hypotensive activity. In another study, it is revealed that hexanoic celery seed extract exerts its hypotensive effects through its bradycardic and vasodilatory properties through Ca^+2^ channel blocking action in endothelial and non-endothelial pathways and particularly by interference with the extra or intracellular calcium.^[93]^ Ko et al^[44]^ showed that apigenin inhibited the contraction of aortic rings caused by cumulative concentrations of calcium in a high potassium medium, with an IC50 of about 48 μM. It also inhibited norepinephrine-induced phasic and tonic contraction in a concentration-dependent manner. They showed that the mechanism of the apigenin effect was related neither cAMP nor to cGMP. The relaxing effect of apigenin on rat thoracic aorta was mainly mediated by suppressing the Ca^+2^ influx through both voltage- and receptor-operated calcium channels. Jorge et al^[94]^ investigated the vasorelaxant effect of organic extracts from *A graveolens*. Their ex vivo method using rat aortic rings with and without endothelium precontracted with norepinephrine showed concentration-dependent relaxation by dichloromethane and ethyl acetate extracts mediated probably by calcium antagonism. In another investigation, it was revealed that the extract of *A graveolens* seed has a relaxation effect on vascular smooth muscle mainly by inhibition of voltage- and receptor-dependent Ca^+2^ channels in vascular smooth muscle cells and may partly by inhibition the release of calcium from the intracellular store in vascular smooth muscle cells beside inhibition of voltage-dependent K^+^ channels.^[95]^ Petroleum ether extract of seeds of *A graveolens* has been evaluated in pigs and showed antispasmodic effects at the dose-dependent manner on histamine-induced contraction in guinea pig’s ileum.^[96]^ In one other study, contractility of isolated atria of the rats in exposure to celery aqueous (decreased the rate of contractions by 12.88 ± 2.74% and amplitude by 8.73 ± 0.89%) and ethanol (inhibited the rate of atria contractions by 34.26 ± 5.69% and amplitude by 25.40 ± 3.61%) extracts exhibited a negative chronotropic and inotropic actions.^[97]^

### 4.3. Antihypertensive component

In an in vivo investigation, the effects of hexanoic extract on systolic, diastolic, mean arterial blood pressure, and heart rate were evaluated in a rat model. Its results indicated that hexanoic extract significantly decreased the systolic, diastolic, mean arterial blood pressure, and heart rate in normotensive and hypertensive rats.^[93]^ In an experimental fructose-induced hypertension model in rats, hydro-alcoholic celery leaf extract reduces SBP, cholesterol, triglyceride, low-density lipoprotein, and very low-density lipoprotein.^[98]^ Chai et al^[99]^ evaluated the effects of aqueous extract of *A graveolens* root on blood pressure and underlying mechanisms in renal hypertension two-kidney one clip rats. They showed that *A graveolens* root extract significantly had increased superoxide dismutase activity, lower malondialdehyde content, and significantly decreased plasm, and endothelin levels. Its high dose also significantly lowered angiotensin levels. In another investigation, the effects of aqueous and ethanol extracts of celery on the mean blood pressure of anesthetized rabbits showed intravenous administration of aqueous extracts induced the least hypotensive effects (14.35 ± 2.94%) versus the ethanol extract caused the most significant fall in the blood pressure (45.79 ± 10.86%). These hypotensive effects were partially blocked by atropine.^[97]^ In a before-after clinical trial on 37 hypertensive patients, 6 g powder of *A graveolens* seeds showed a significant decrease from baseline (171.35/94 mm Hg to 154.30/89.6 mm Hg).^[88]^ In a double-blind randomized clinical trial in patients with mild to moderate hypertension, *A graveolens* plus another medicinal plant was administered and compared with the group receiving amlodipine for 12 weeks. Their results showed that the herbal complex lowered systolic and diastolic blood pressure equivalent to amlodipine with no side effects.^[100]^ In another clinical pre and post-test study on 30 adult males, it was demonstrated that drinking celery ethanol extract decreased blood pressure significantly from 116.02/ 74.79 mm Hg to 109.40/70.20 mm Hg.^[101]^ Extracts and constituents of celery have been reported to the lower arterial pressure in humans, possibly by lowering levels of circulating catecholamines and decreasing vascular resistance.^[91]^ The summary of *A graveolens* effects is listed in Table 3.

**Table 3:** Potential effects of *A graveolense* in treatment of CHF.

Study	Therapeutic component	Dose/active constitute/extract	Type of study	Major findings/mechanism
Zhang et al^[94]^	Vasodilator	Apigenin	In vitro rat aortic rings	Relaxed endothelium-intact aortic rings by influx and release of Ca^+,2^ NO and cGMP
Tashakori-sabzevar et al^[95]^	Vasodilator	Hexanic celery seed extract (2.5-12.5 mg/kg)	In vivo rats	Ca^+2^ channel blocking activity in endothelial and non-endothelial pathways
Ko et al^[46]^	Vasodilator	Apigenin	In vitro rat aortic rings	Inhibited the contraction of aortic rings by suppressing the Ca^+2^ influx through both voltage- and receptor-operated calcium channels
Jorge et al^[96]^	Vasodilator	Dichloromethane and ethyl acetate extracts	In vitro rat aortic rings	Made relaxation by calcium antagonism effect
Niazmand et al^[97]^	Vasodilator	Hydroalcoholic extract of seed	In vitro rat isolated aorta	Vascular smooth muscle relaxation by inhibition of voltage- and receptor-dependent Ca^+2^ channels
Ahmed et al^[98]^	Vasodilator	Petroleum ether extract of seeds	In vitro guinea pig’s ileum	Antispasmodic effects by inhibiting histamine induced contraction
Tashakori-sabzevar et al^[95]^	Antihypertensive	Hexanic celery seed extract (2.5–12.5 mg/kg)	Rats	Decreased the systolic, diastolic, mean arterial blood pressure and heart rate in normotensive and hypertensive rats
Dianat et al^[100]^	Antihypertensive	Hydro-alcoholic celery leaf extract	In vivo fructose-induced hypertension rats	Reduced SBP, cholesterol, triglyceride, LDL and VLDL
Chai et al^[101]^	Antihypertensive	Root extract	In vivo renal hypertension two-kidney one clip (2K1C) rats	Increased SOD activity, lower MDA content, decreased angiotensin levels
Brancovic et al^[99]^	Antihypertensive	Aqueous and ethanol extracts of celery	In vivo rabbits	Lowered the mean blood pressure (ethanolic extract more than aqueous extract)
Gharouni et al^[90]^	Antihypertensive	6 g powder of seeds	Before-after clinical trial	Significant decrease in BP from baseline in 37 hypertensive patients
Supari^[102]^	Antihypertensive	Celery plus another herbal medicine for 12 wk	Double blind randomized clinical trial patients with mild to moderate hypertension	Lowered systolic and diastolic blood pressure equivalent amlodipine with no side effect

cGMP = cyclic guanosine monophosphate, CHF = chronic heart failure, LDL = Low-density lipoprotein, SBP = systolic blood pressure, SOD = superoxide dismutase, VLDL = very low-density lipoprotein.

## 5. Peganum harmala

*Peganum harmala L.*, used in traditional medicine from ancient times, is considered an important medicinal plant for the treatment of a variety of human ailments.^[102,103]^ Its antihypertensive effect^[30–32,64,89]^ and diuretic effect^[30,31,64]^ were suggested in traditional medicine references. *P harmala* is one of the most frequently used medicinal plants to treat hypertension and cardiac disease worldwide.^[104]^ Many pharmacological studies suggest an antioxidant and free radical scavenging effect of *P harmala*.^[105]^

### 5.1. Chemical constituents

Wang et al^[106]^ investigated the constituents this herb and detected alkaloids from *P harmala* seeds in 2 pairs of unique racemic pyrroloindole alkaloids, (±)-peganines A-B (1-2); 2 rare thiazole derivatives, peganumals A-B (3-4); six new β-carboline alkaloids, pegaharmines F-K (5-10); and 12 known analogs. Harmaline (1-methyl-7-methoxy-4,9-dihydro β-carboline),^[107]^ harmine, and harmalol^[108,109]^ are the most essential compounds in seeds of *P harmala*.

### 5.2. Vasodilator and Ca channel blocker component

Berrougui et al^[110]^ described the mechanisms involved in the vasorelaxant effect of harmine, and harmaline. They showed that these alkaloids induce a dose-dependent relaxation in the aorta precontracted with noradrenaline or KCl. This mechanism in harmaline was mediated by eNOS (Endothelial Nitric oxide synthase expression) synthetase, cyclo-oxygenase and alpha-adrenoreceptors pathways but not in harmine. However, inhibiting phosphodiesterase affects both the harmaline and harmine responses. L-type voltage-dependent Ca^+2^ channels in endothelium-intact aortic rings were mediated in the vasorelaxant result of both compounds. So, it was revealed that the vasorelaxant effect of harmaline but not harmine is related to its action on the prostacyclin pathway and on the endothelial cells to release NO. However, both alkaloids can act as blockers of volatile organic compounds as inhibitors of phosphodiesterase, resulting in an increasing of the second messenger (cAMP and cGMP) levels and, finally reducing the free radicals in tissues. Shi et al^[111]^ showed vasorelaxant activities of 3 compounds of *P harmala* in isolated rat thoracic aorta by phenylephrine or KCl with the rank order of potency of harmine > harmaline > harmalol. They also stated that the vasorelaxant effects of harmine, and harmaline (but not harmalol = vasorelaxant effect was not endothelium-dependent) were related to NO release involvement and all of them inhibited phenylephrine-induced contractions, in a noncompetitive manner. They also showed the interaction with cardiac alpha1-adrenoceptors with comparable affinities in all 3 compounds but only harmine weakly interacted with the cardiac 1,4-dihydropyridine binding site of L-type Ca^+2^ channels. Astulla et al^[108]^ reported a quinazoline alkaloid, vasicinone (3) in *P harmala* seed extract with a vasorelaxant activity against phenylephrine-induced contraction of isolated rat aorta. In another study, it is shown that the decreased blood pressure in *P harmala* might be caused by depletion in peripheral vascular resistance and non-dependent endothelium manner accomplished by vascular smooth muscle relaxation.^[112]^ In another study,^[113]^ the activity of methanolic extract from the seeds of *P harmala* on the vascular smooth muscle in rat aorta showed that the extract exerts a vasodilatory effect not related to the presence of endothelium but mainly due to the inhibition of cyclic AMP phosphodiesterase. Karaki et al^[114]^ examined the effects of harmaline and other harmala alkaloids on the contractions induced in the vascular smooth muscle of rabbit aorta and intestinal smooth muscle of tenia isolated from the guinea-pig cecum. They showed that harmaline inhibited the sustained contraction induced by K + and inhibited the sustained contraction induced by noradrenaline. They showed the order of the inhibitory potency as 6-methoxyharman = harmine > harmaline = 2-methylharmine = harmane > 6-methoxyharmalan > harmalol = harmol for the contractions induced by high K + in the aorta and tenia and by carbachol in tenia, and 2-methylharmine > 6-methoxyharman > 6-methoxyharmalan = harmol = harmalol = harmane > harmine > harmaline for the contraction induced by noradrenaline in the aorta. This indicated that harmaline inhibits a different type of Ca^+2^ channel. Harman, another constituent of *P harmala,* showed that dose-dependently produced transient hypotension and long-lasting bradycardia in pentobarbital-anesthetized rats via interaction with the cardiac α1-adrenoceptors, brain 5-HT_2_ receptors, and cardiac 1,4-dihydropyridine binding site of L-type Ca^[2]+^ channels.^[115]^ It had been also showed that the aqueous extract of *P harmala* had antispasmodic, anticholinergic, antihistaminic, and antiadrenergic effects using isolated segments of the intestine, trachea, and aorta in vitro.^[116]^

### 5.3. Diuretic component

Al-Saikhan and Ansari^[102]^ assessed the effect of methanolic extract of *P harmala* in comparison with a well-known diuretic drug furosemide in Wistar albino rats. It showed that in 3 different doses (150, 300, and 450 mg/kg), it could significantly increase urine output and urinary electrolyte excretion in a dose-dependent manner.

### 5.4. Inotropic component

Aarons et al^[117]^ showed that each of the 3 harmala alkaloids, harmine (diminish systolic arterial blood pressure and total peripheral vascular resistance), harmaline, and harmalol, lowered heart rate and increased pulse pressure, peak aortic flow, and myocardial contractile force entire normotensive anesthetized dogs. A direct negative chronotropic effect of harmala alkaloids was suggested by observations of bradycardia in the isolated perfused rat heart and in the intact dog. It is shown in another study^[118]^ that quinidine and harmine of *P harmala* can prolong repolarization time dose-dependently in isolated guinea-pig atria. The depolarization velocity was considerably reduced by quinidine, while it was little affected by harmine.

### 5.5. Antihypertensive component

It is shown that^[119]^ the active compound of *P harmala*, harmaline in its methanolic extract, inhibited 48.7% the ACE activity at 1 μg and was very close to the inhibition observed with the standard drug, captopril at 49.1% (1 μM). It is shown that ACE inhibitory effect percent of its aqueous and ethanolic extracts were 72 and 84, respectively.^[48]^ In another study, its inhibitory effect on germinal the ACE enzyme was 70% for ethanolic extract and on somatic ACE enzyme 84% and 72% for ethanolic and aqueous extracts, respectively.^[69]^ In an in vivo study on isolated thoracic aorta rings in male rats it was shown that an aqueous extract of *P harmala* significantly decreased systolic blood pressure from 140 ± 5 mm Hg to 101 ± 1 mm Hg.^[112]^ In another experimental study, in normotensive anesthetized rats, intravenous administration of harmalol produced a dose-dependent fall in blood pressure and heart rate.^[120]^ The summary of *P harmala* effects is listed in Table 4.

**Table 4:** Potential effects of *P harmala* in treatment of CHF.

Study	Therapeutic component	Dose/active constitute/extract	Type of study	Major findings/mechanism
Berrougui et al^[108]^	Vasodilator	Harmaline	In vitro rat aorta	Vasorelaxant effect mediated by prostacyclin pathway and on the endothelial cells to release
Shi et al^[112]^	Vasodilator	Harmine, harmaline, harmalol	In vitro isolated rat thoracic aorta	Vasorelaxant activities harmine > harmaline > harmalol not endothelium-dependent related to NO release and interaction with cardiac alpha1-adrenoceptors
Astulla et al ^[113]^	Vasodilator	Vasicinone ^[3]^	In vitro isolated rat aorta	Vasodilator effect
Karaki et al^[116]^	Vasodilator	Harmaline	In vitro smooth muscle of rabbit aorta and intestinal smooth muscle of tenia isolated from guinea-pig cecum	Inhibited the sustained contraction induced by K + and inhibited the sustained contraction induced by noradrenaline inhibit different type of Ca^+2^ channel
Al-Saikhan & Ansari^[118]^	Diuretic	Methanolic extract of *P harmala* 150, 300 and 450 mg/kg	In vivo Wistar albino rats	Significantly increase urine output and urinary electrolyte excretion dose dependently
Aarons et al^[119]^	Inotropic	Harmine harmaline, and harmalol	In vivo dogs In vitro isolated perfused rat heart	Lowered heart rate increased pulse pressure, peak aortic flow, and myocardial contractile force
Iven et al^[120]^	Inotropic	Quinidine and harmine	In vitro isolated guinea-pig atria	Prolong repolarization time dose-dependently
Farrokhfal et al^[114]^	Antihypertensive	Aqueous extract of *P harmala*	In vitro isolated rat thoracic aorta rings	Significantly decreased systolic blood pressure
Gilani et al^[121]^	Antihypertensive	Harmalol	In vivo normotensive anaesthetized rats	Dose-dependent fall in blood pressure and heart rate

CHF = chronic heart failure.

## 6. Citrus aurantifolia swings

According to World Health Organization’s recent report, citrus fruits offer protection against cardiovascular diseases by reducing homocysteine levels. Orange fruit contained vitamin C, carotenoids and flavonoids, which were cardio-protective. The cholesterol-lowering effect of orange was produced by limonene.^[122]^ Citrus aurantifolia fruit peels have long been a source of herbal products, especially in Chinese medicine^[121]^ and traditional medicine (diuretic, cardiotonic, and antihypertensive effects).^[30–32,64,89]^

### 6.1. Chemical constituent

Citrus (Rutaceae) are rich source of many exciting constituents, such as volatile oils, steroids, limonoids, flavonoids, coumarins, and pectins of different bioactivities, and nutritional values.^[123]^ Psoralene, bergapten, isopimpinellin, imperatorin, myricetin, rutin, and hesperidin are the compounds of *C aurantifolia* peels.^[124]^ The ingredients of Citrus aurantifolia peel essential oil have been investigated to be limonene, alpha-terpineol, gamma-terpinene, terpinolene, linalool, and cineole.^[121]^ In another study based on analysis by high-performance liquid chromatography, the most abundant flavonoids found in *C aurantifolia* extracts were apigenin, rutin, quercetin, kaempferol, and nobiletin.^[125]^

### 6.2. Inotropic component

Souza et al showed that the aqueous extract of *C aurantifolia* induced both negative inotropic and chronotropic effects on the heart contractile activity in rat aorta.^[126]^

### 6.3. Antihypertensive component

It is shown that the methanol extract of *C aurantifolia*, administered at the dose of 0.75 mg orally, in rats significantly reduced systolic blood pressure, mean blood pressure, diastolic blood pressure, heart rate, and body weight in both normotensive and hypertensive experimental models when compared to control groups.^[127]^ In another study, it was demonstrated aqueous extract of *C aurantifolia* produced a dose-dependent and significant decrease in rabbit blood pressure of adrenalin-evoked hypertension.^[126]^ *C aurantifolia* juice inhibited ACE activity in a dose-dependent manner and also exhibited antioxidant activities as typified by their ferric reducing power and radicals (DPPH·, ABTS·, OH·, and NO·) scavenging abilities, as well as inhibition of Fe - and sodium nitroprusside-induced lipid peroxidation, in rat’s liver in vitro.^[128]^ Hashemipour et al in a triple-masked randomized controlled trial, assessed *C aurantifolia* peels on the cardiometabolic risk factors and markers of endothelial function in adolescents with excess weight. However, their results showed that the change of none of the cardiometabolic risk factors, such as SBP (systolic blood pressure) and (diastolic blood pressure) DBP, was significant in comparison with the placebo group. SBP was significantly decreased in the intervention group in a before-after analysis.^[129]^ Siti et al^[130]^ determined the effects of Citrus leaf extract on blood pressure, blood pressure-regulating enzymes and mediators, as well as aortic histomorphometry in heated palm oil induced-hypertension Sprague Dawley rats and showed that it can reduce the heated oil-raising effect on blood pressure, plasma TBARS, thromboxane and angiotensin-1 converting enzyme in five-times-heated palm oil and increase serum heme oxygenase-1. It also reduced the increase in aortic intima-media thickness, intima-media area and circumferential wall tension. It is shown that the aqueous extract of *C aurantifolia* can inhibit the ACE enzyme by 67% and ethanolic extract by 35%^[48]^ and inhibit germinal ACE by ethanolic and aqueous extracts by 53.3% and 13.3% besides inhibiting somatic ACE by ethanolic and aqueous extracts by 35% and 67%, respectively in another study.^[69]^

### 6.4. Vasodilator component

In an experimental study, aqueous extract of *C aurantifolia* induced a dose-dependent relaxation of contractions produced by adrenalin or by KCl, and evoked vasorelaxant effects were abolished by removal of the endothelium layer or by pretreatment with L-NAME.^[126]^ Ahounou et al evaluated the spasmolytic properties of the mixed aqueous extract of *Aframomum melegueta* and *C aurantifolia* on isolated rat trachea mediated by acetylcholine receptor inhibition.^[131]^ However, this study related to a mixture of extracts and is not only *C aurantifolia*. The summary of *Citrus aurantifolia* effects is listed in Table 5.

**Table 5:** Potential effects of *Citrus aurantifolia swings* in treatment of CHF.

Study	Therapeutic component	Dose/active constitute/extract	Type of study	Major findings/mechanism
Souza et al^[128]^	Inotropic	aqueous extract of *C aurantifolia*	In vitro rat aorta	Negative inotropic and chronotropic effects on the heart contractile activity
Akhtar^[129]^	Antihypertensive	methanol extract of *C aurantifolia* 0.75mg orally	In vivo rats	Significantly reduced systolic blood pressure, mean blood pressure, diastolic blood pressure, heart rate and body weight in both normotensive and hypertensive experimental models
Souza et al^[128]^	Antihypertensive	aqueous extract of *C aurantifolia*	In vivo rabbit	Dose-dependent and significant decrease in blood pressure of adrenalin-evoked hypertension
Hashemipour et al^[131]^	Antihypertensive	*C aurantifolia* peels	Triple-masked randomized controlled trial in adolescents with excess weight	SBP was significantly decreased in intervention group in a before-after analysis
Siti et al^[132]^	Antihypertensive	Citrus leaf extract	In vivo palm oil induced-hypertension Sprague Dawley rats	Reduce blood pressure, thromboxane and angiotensin-1 converting enzyme
Souza et al^[128]^	Vasodilator	aqueous extract of *C aurantifolia*	In vivo rabbit	Dose dependent relaxation of contractions produced by adrenalin

CHF = chronic heart failure, SBP = systolic blood pressure.

### 6.5. Other beneficial components

In a triple-masked randomized trial, *C aurantifolia* was assessed on endothelium function in adolescents with excess weight and showed significantly higher flow-mediated dilatation in this group rather than in placebo group.^[132]^

## 7. Citrus aurantium

Extracts of *Citrus aurantium L*. unripe fruits have been used for centuries in traditional medicine.^[133]^ There is thus a history of benign human consumption of *C aurantium* fruit. It has pharmacological applications that its flowers have been used as a sedation and heart tonic in traditional medicine.^[134]^ The Hydrolate of its flowers has been used in traditional medicine as a heart tonic.^[32,135]^ In traditional resources, it is indicated the flower of this herb had diuretic^[31,32,64]^ and cardiotonic effects^[30,89]^ and had no antihypertensive effect.

### 7.1. Chemical constituents

The peel of *C aurantium L*. contains limonene as the principal essential oil constituent, flavonoids, hesperidin, neohesperidin, naringin, and tangaretin and it is often used in marmalade production.^[136]^ Limonene and β-myrcene were reported as the major components of the peel oil while linalool, linalyl acetate and α-terpineol predominate in the leaf oil (petitgrain).^[137]^ The most important active constituent of the plant is the sympathomimetic compound synephrine.^[138]^

### 7.2. Vasodilator and Ca channel component

*C aurantium* extract can increase the L-type calcium current in guinea pig ventricular myocytes significantly in a concentration-dependent manner and promote the opening of calcium channel.^[139]^ It is shown that^[140]^ Neroli (the essential oil of *C aurantium*) is a well-characterized alleviative agent used to treat cardiovascular symptoms. In aortic rings of mice precontracted with prostaglandin F2 alpha, neroli induced vasodilation by inducing nitric oxide synthase and inhibiting extracellular Ca^+2^-dependent, depolarization-induced contraction in a dose-dependent manner. It is indicated that the endothelial component of neroli-induced vasodilation is partly mediated by the NO-sGC pathway. In contrast, the smooth muscle component involves modulation of intracellular Ca^+2^ concentration through inhibition of cation channel-mediated extracellular Ca^+2^ influx and store-operated Ca^+2^ release mediated by the Ryanodine receptor signaling pathway. In another experimental study, it was revealed that cumulative concentrations of the extract of *C aurantium* decreased KCl, oxytocin, and barium chloride-induced uterine contractions of rats, dose-dependently via calcium influx blockade without either β-adrenoceptors nor opioid receptors involvement.^[141]^ Huang et al^[142]^ have shown that at a dose of 1 mg/kg orally twice a day in 2 models of portal hypertensive rats, p-synephrine significantly ameliorated the hyperdynamic effects. In another study, crude aqueous extract of *C aurantium* or pure synephrine reduced portal pressure both in rats with surgically induced portal hypertension and sham-treated rats.

### 7.3. Antihypertensive component

Aqueous and ethanolic extracts of *C aurantium* can inhibit the ACE enzyme, 60% and 56%, respectively.^[48]^ In another assessment, its aqueous extract was inhibited by 86.6% the germinal ACE enzyme and ethanolic and aqueous extracts were inhibited by 56% and 60% somatic ACE enzyme, respectively.^[69]^ There are no antihypertensive effects of the *C aurantium* or its ingredients on lowering blood pressure. However, we mentioned some similar studies in portal hypertension and the effect of the herbs. The *C aurantium* extract had a more significant impact in the portal hypertension model and the efficacy of vasoconstrictors to reduce portal pressure probably contributed to their constrictive actions in the splanchnic arterial circulation.^[143]^ Based on the receptor binding studies, p-synephrine would be expected to exert little vasoconstriction and, therefore, little or no effect on blood pressure as compared to m-synephrine or norepinephrine.^[144]^ The vast majority of human clinical studies have noted that p-synephrine and bitter orange extract alone or in combination with caffeine and other ingredients do not affect on blood pressure or heart rate.^[145–149]^

### 7.4. Inotropic component

Studies of the flowers of this natural medicine have reported that some constituents exhibited antioxidant activity by flavonoids containing and the aqueous extract showed an inotropic impact. The *C aurantium* extract had an impact on hypertension.^[132]^ Based on receptor binding studies using human and animal cells, m-synephrine exerts its effects on α-1, β-1, and β-2 adrenergic receptors,^[150,151]^ resulting in increased blood pressure, and heart rate effects. Hibino et al^[152]^ examined the ability of p-synephrine to constrict isolated rat aorta at different doses. It concluded that the constrictor effects were exerted via α-1 adrenoreceptors and serotonergic (5-HT and 5-HT) receptors. The results suggested that p-synephrine might produce vasoconstriction but only at concentrations many times above the blood levels achieved under normal conditions of oral usage. In another investigation,^[153]^ the effects of *C aurantium* and one of its active ingredients, N-methyl-tyramine (II), on vascular resistance were studied and showed that the cardiovascular effects of the extract and main active ingredient resulted from the liberation of intrinsic adrenergic mediator, which shares a direct agonist action on α-receptors. The summary of *C aurantium* effects is listed in Table 6.

**Table 6:** Potential effects of *Citrus aurantium* in treatment of CHF.

Study	Therapeutic component	Dose/active constitute/extract	Type of study	Major findings/mechanism
Arbo et al^[141]^	Vasodilator	*C aurantium* extract	In vitro guinea pig ventricular myocytes	Promote the opening of calcium channel
Kang et al^[142]^	Vasodilator	Essential oil of *C aurantium*	In vitro aortic rings of mice	Induced vasodilation by inducing nitric oxide synthase and inhibiting extracellular Ca^+2^-dependent depolarization-induced contraction dose-dependently
Ahangarpour et al^[143]^	Vasodilator	Extract of *C aurantium*	In vitro Rat uterine	Decreased induced uterine contractions dose-dependently via calcium influx blockade without neither β-adrenoceptors nor opioid receptors involvement
Fugh-Berman et al^[135]^	Inotropic	The aqueous extract	In vitro	Inotropic effect
Hibino et al^[154]^	Inotropic	p-synephrine	In vitro isolated rat aorta	Constrict isolated rat aorta via α-1 adrenoreceptors and serotonergic (5-HT and 5-HT) receptors
Huang et al^[145]^	Antihypertensive	*C aurantium* extract	In vivo rats	Reduce portal pressure probably due to splanchnic arterial circulation

CHF = chronic heart failure.

## 8. Allium sativum

Garlic has been used for many years in the traditional medical practice of various cultures to reduce the risk of several diseases, mainly of the cardiovascular system. It is generally believed that garlic exerts preventative rather than curative effects.^[154]^ Medicinal uses date back to 1550 BCE when it was used as a remedy for heart disease, headaches, and tumors. It has also been used as an aphrodisiac to improve sexual performance and desire and as a cure-all for everything from hemorrhoids to snake bites.^[155]^

### 8.1. Chemical constituents

The contents of the extract include sulfur active principles mainly in the form of cysteine derivatives such as s-alkyl cysteine and sulfaxides, which decompose into a variety of thiosulfinates and polysulfides by the action of an enzyme allinase on extraction.^[156,157]^ When garlic is chopped or crushed, the allinase enzyme present in garlic is activated and acts on alliin (present in intact garlic) to produce allicin. Other essential sulfur-containing compounds present in garlic homogenate are allyl methyl thiosulfonate, 1-propenyl allyl thiosulfonate, and γ-L-glutamyl-S-alkyl-L-cysteine.^[158]^ Allicin is the odoriferous substance of garlic which was discovered by Cavallito et al, while investigating the antibacterial properties of garlic. This compound is widely considered one of the most important biologically active compounds produced by garlic and was shown to be 2-propenyl-2-propene-thiosulfinate, commonly known diallyl thiosulfinate.^[159]^

### 8.2. Inotropic component

Garlic dialysate decreased the positive inotropic and chronotropic effects of isoproterenol in a concentration-dependent manner. β-receptor blocking action of garlic was also suggested by Martin et al.^[160]^ The positive inotropic and chronotropic induced by isoproterenol were partially antagonized by preincubation of the rat atria with the garlic dialysate. The electrocardiogram showed a regular sinus bradycardic rhythm in garlic dialysate-fed anesthetized rat.

### 8.3. Vasodilator component

In an experimental study,^[161]^ the effect of oral administration of allium ursinum on thoracic aorta contractile responsiveness in diabetic rats was assessed. The results showed that it could attenuate the contractile responsiveness of the vascular system in diabetic rats. Garlic also inhibited endothelin-1-induced contraction in a dose-dependent manner in isolated rat pulmonary arteries.^[162]^ Garlic modulates the production and function of both endothelium-derived relaxing and constricting factors, and this may contribute to its protective effect against hypoxic pulmonary vasoconstriction.^[162]^ Another study reported that the pulmonary vasodilatory effect of allicin is independent of the synthesis of NO, ATP-sensitive (K+) channel and activation of cyclooxygenase enzyme.^[157]^ Garlic juice inhibited norepinephrine-induced contractions of rabbit and guinea pig aortic rings. It also inhibited the force of contraction of isolated rabbit hearts in a concentration-dependent manner.^[163]^ Another vasorelaxation effect of garlic is mediated through H2S production liberated from alliin and the enzyme alliinase.^[164]^ Ashraf et al^[170]^ indicated in their experimental study that endothelium-modulated vasorelaxation of garlic is partly mediated through EDHFs (Endothelium-derived hyperpolarizing factor) and cycloxygenase pathways. However, relaxing factor(s) other than NO, mediated through cGMP, has a significant role in the vasorelaxant response of garlic.

### 8.4. Antihypertensive component

The gamma-glutamylcysteines are the compounds in garlic that may lower blood pressure, as indicated by their ability to inhibit the angiotensin-converting enzymes in vitro.^[166]^ In recent studies, aqueous extract of *A sativum* inhibit germinal and somatic ACE enzyme by 70% and 76%, and ethanolic extract inhibited the germinal and somatic ACE enzymes by 60% and 68%.^[69]^ In other studies, the ACE inhibitory and angiotensin inhibition effect of garlic was confirmed.^[167,168]^ Reinhart et al, in a meta-analysis, evaluated the antihypertensive effects of garlic and included 10 trials in their analysis. Their results showed that garlic reduced SBP by 16.3 mm Hg (95% CI 6.2–26.5) and DBP by 9.3 mm Hg (95% CI 5.3–13.3) compared with placebo in patients with elevated SBP.^[169]^ García-Trejo et al^[170]^ showed that allicin treatment attenuated hypertension and improved the renal and cardiac dysfunctions; decreased the vascular reactivity to angiotensin II, AT1R overexpression, and preserved morpho-metric parameters; also down-regulated Keap1 and increased Nrf2 expression, up-regulated the anti-oxidant enzymes, and reduced oxidative stress in a rat model of CKD. In another study, it was shown that its aqueous and ethanolic extracts inhibited ACE enzyme by 76% and 68%, respectively.^[48]^ Allicin showed blood pressure-lowering effects in different animal studies.^[171,172]^ Oron-Herman et al,^[173]^ showed that treatment with both low dose and high doses of S-allylmercaptocaptopril, the synthetic product of allicin (5 mg/kg/d), lowered SBP. In animal models, intravenous injection of garlic extracts made a mild decrease in both systolic and diastolic pressures.^[174,175]^ Oral use of garlic decreased experimentally-induced hypertension and returned blood pressure to the normal range. For example, 2.5 to 25 mg per kg of alcoholic garlic extract reduced blood pressure by 100 to 50 mm Hg.^[176]^ Single and multiple doses of aqueous garlic extract reduced thromboxane B2 and prostaglandin E2 levels and thereby reduced hypertension in the “2 kidney 1-clip” model of hypertension in rat.^[177]^ The antihypertensive effect of garlic in these studies has been repeatedly confirmed. There are many clinical studies in humans with different doses, in the range of 0.6 to 2.4 g in other studies, and showed significant decreases in blood pressure in various types of garlic preparation.^[178–183]^ In a clinical trial, Ashraf et al^[184]^ showed in 210 patients that consumption of garlic could significantly decrease both SBP and DBP in garlic groups rather than atenolol. Beta-adrenoceptor blocking action of garlic is seen in vitro study.^[185]^ The summary of *Allium sativum* effects is listed in Table 7.

**Table 7:** Potential effects of *A sativum* in treatment of CHF.

Study	Therapeutic component	Dose/active constitute/extract	Type of study	Major findings/mechanism
Martin et al^[162]^	Inotropic	Total extract	In vitro, rat atria In vivo, dogs	Positive inotropic and Chronotropic effects by β-receptor blocking
Kaye et al^[164]^	Vasodilator	Allicin	In vitro rat	Pulmonary vasodilatory effect independent of the synthesis of NO, ATP-sensitive (K+) channel, and by activation of cyclooxygenase enzyme
Aqel et al^[165]^	Vasodilator	Garlic juice	In vitro rabbit and guinea pig aortic rings	Inhibited norepinephrine-induced contractions and inhibited the force of contraction of isolated rabbit heart in a concentration-dependent
Ashraf et al^[167]^	Vasodilator	Total extract	In vitro	Vasodilator effect mediated through EDHFs and cycloxygenase pathways
Reinhart et al^[171]^	Antihypertensive	Garlic preparations	Meta-analysis of clinical trials	Reduced SBP and DBP
García-Trejo et al^[172]^	Antihypertensive	Allicin	In vivo a rat model of CKD	Attenuated hypertension and improved the renal and the cardiac dysfunctions decreased the vascular reactivity to angiotensin II, AT1R overexpression
Oron-Herman et al^[175]^	Antihypertensive	S-allylmercaptocaptopril (synthetic product of allicin) 5 mg/kg/day	In vivo rats	Lowered SBP
Chanderkar and Jain^[178]^	Antihypertensive	Alcoholic garlic extract	In vivo	Reduced experimentally induced hypertension, bringing blood pressure back to the normal range
Ashraf et al^[186]^	Antihypertensive	Garlic preparation oral consumption	Clinical trial in 210 patients	Decrease both SBP and DBP

CHF = chronic heart failure, DBP = diastolic blood pressure, EDHF = endothelium-derived hyperpolarizing factor, SBP = systolic blood pressure.

### 8.5. Other beneficial components

In an experimental investigation, it was shown that allicin attenuated LV mass, posterior wall thickness and LV end-diastolic diameter and increased fractional shortening, and EF in the Angiotensin-II-induced hypertrophic rats compared to the untreated Ang-II rats. It also decreased the accumulation of interstitial collagen and collagen I/III, the levels of reactive oxygen species, protein carbonyl and TBARS. It increased GPx activities, mRNA expression and protein levels of Nrf2, NQO1, and γ-GCS.^[186]^ Castro et al,^[168]^ showed that allyl methyl sulfide, and diallyl sulfide from garlic prevented aortic smooth-muscle cell angiotensin II-stimulated cell-cycle improvement and migration by inhibiting the cell cycle inhibitor p27Kip1 (p27) down-regulation, and the decrease of extra-cellular signal-regulated kinase 1/2 phosphorylation.

## 9. Cerasus avium

*Cerasus avium (C avium*) or *Prunus avium* stems have been highly utilized in traditional medicine. After having been dried and boiled, *C avium* stem is used for treatment. Its fruit stalks are sold by herbal druggists in Iran and are used as a decoction to relieve renal stones and hypertension.^[187]^ The diuretic effect of the stalks has been reported in traditional medicine, documents due to the high content of flavonoids and potassium.^[31,32,188]^ In different resources of traditional medicine diuretic,^[30,64,89]^ cardiotonic,^[30]^ and antihypertensive^[30–32,64,89]^ effects are mentioned.

### 9.1. Chemical constituents

*C avium* stems have different compounds which are antioxidant and including caffeic acid, ferulic acid, syringic acid, ellagic acid, quercetin, α-tocopherol, pyrogallol, p-hydroxybenzoic acid, vanillin, p-coumaric acid, gallic acid, and ascorbic acid.^[189]^ One of the highest percent compounds in *C avium* is quercetin.^[190]^

### 9.2. Diuretic component

Hooman et al^[191]^ evaluated the diuretic effect of the cherry stalks in healthy volunteers and showed that administration of capsules of cherry stalks at an equivalent dose of 2.0 g of the plant per person led to an increase in the mean of urine calcium, sodium, chloride, and urine volume increased without any adverse reaction. They indicated that increased mild urine volume confirmed the claimed diuretic effect of the herb. The diuretic effect of *C avium* stalk in Turkish folk medicinal plants is mentioned.^[192]^ In an experimental study, it was shown that *C avium* stem has a therapeutic effect on calcium oxalate stones in rats with nephrolithiasis and reduces the number of calcium oxalate deposits which was because of its diuretic effect.^[193]^

### 9.3. Vasodilator component

Repasi and Frank^[194]^ evaluated the effect of Novicardine, the acetonic extract of *P avium* peduncle on isolated heart preparations in the organ bath. They showed that it improves the contraction force of heart muscle by some 20% to 25%, and at the same time does not exert influence, on the basic electrophysiological parameters and does not cause significant changes in heart rate and have a negative inotropic effect. It is shown that in isolated rat aorta quercetin produced a vasodilator effect that seems to be mainly related to the inhibition of protein kinase C.^[195]^ In another experimental study, quercetin produced endothelium-independent vasodilator effects in rat aorta, rat mesenteric arteries, rat portal vein, and porcine coronary arteries.^[196]^ Quercetin has been shown to have vasodilator effects in vitro using isolated rat arteries independent of the endothelium.^[197–199]^

### 9.4. Antihypertensive component

In in vitro study, it is demonstrated that the ethanolic extract of *C avium* inhibits germinal and somatic ACE enzymes by 70% in both and its aqueous extract by 100% and 77%, respectively.^[69]^ In another study, it inhibited ACE enzyme in the aqueous extract by 77% and ethanolic extract by 70%.^[48]^ It is shown that quercetin can lower blood pressure in different models of hypertensive models, like spontaneously hypertensive,^[200]^ Dahl salt-sensitive rats^[201]^ high-fat, high-sucrose diet model,^[202]^ models deficient in NO,^[203]^ Angiotensin-I-infused hypertension.^[204]^ Supplementation of the diet with quercetin aglycone has been shown to decrease blood pressure in hypertensive individuals (Decreases in systolic [−7 ± 2 mm Hg], diastolic [−5 ± 2 mm Hg], and mean arterial blood pressure [−5 ± 2 mm Hg] in subjects with hypertension) after supplementation with 730 mg/d of quercetin for 28 days vs placebo.^[205]^ Two studies by Egert et al,^[206,207]^ found that 150 mg/d of quercetin for 6 weeks decreased blood pressure in overweight and obese prehypertensive individuals. Both studies by Egert et al found statistically significant decreases in systolic pressure of −3 mm Hg. The probable mechanisms of quercetin are a decrease in oxidative stress, interference with the renin-angiotensin-aldosterone system (RAAS), and or improving vascular function in an endothelium-dependent or -independent manner.^[208]^ The summary of *C avium* effects is listed in Table 8.

**Table 8:** Potential effects of *C avium* in treatment of CHF.

Study	Therapeutic component	Dose/active constitute/extract	Type of study	Major findings/mechanism
Hooman et al^[193]^	Diuretic	Capsules of cherry stalks 2.0 g	Clinical trial in healthy volunteers	Increase in the mean of urine calcium, sodium, chloride, and urine volume increased without any adverse reaction
Repasi and Frank^[196]^	Inotropic	Acetonic extract of *P avium*	In vitro isolated heart rat	It improves the contraction force of heart muscle by some 20–25%
^[198]^	Vasodilator	quercetin	In vitro rat aorta	Endothelium-independent vasodilator effects
Egert et al^[208,209]^	Antihypertensive	Quercetin 150 mg/d for 6 wk	Clinical trial in overweight and obese prehypertensive individuals	Significant decreases in systolic pressure

CHF = chronic heart failure.

## 10. Conclusion

In conclusion, any medicinal plants mentioned in this review have many potentials to be a mixed or isolated treatment option for CHF and the efficacy of their quality of life. It is better to find these mechanisms and make a complex treatment according to evidence and historical traditional notes and experiences. Isolation and discovering new drugs for the treatment of CHF is now going on, and based on the recommended medicines, it can be improved and hoped to make newer agents.

## Author contributions

**Conceptualization:** Faeze Keihanian, Mohsen Moohebati, Seyed Ahmad Mohajeri.

**Investigation:** Faeze Keihanian, Mohsen Moohebati, Seyed Ahmad Mohajeri.

**Methodology:** Faeze Keihanian, Seyed Ahmad Mohajeri.

**Writing – original draft:** Faeze Keihanian, Amin Saeidinia.

**Writing – review & editing:** Faeze Keihanian, Mohsen Moohebati, Amin Saeidinia, Seyed Ahmad Mohajeri.
